# Zika virus infection accelerates Alzheimer’s disease phenotypes in brain organoids

**DOI:** 10.1038/s41420-022-00958-x

**Published:** 2022-04-02

**Authors:** Seung-Eun Lee, Hanul Choi, Nari Shin, Dasom Kong, Nam Gyo Kim, Hee-Yeong Kim, Min-Ji Kim, Soon Won Choi, Young Bong Kim, Kyung-Sun Kang

**Affiliations:** 1 Adult Stem Cell Research Center and Research Institute for Veterinary Science, College of Veterinary Medicine, Seoul National University, Seoul, 08826 Republic of Korea; 2 Department of Biomedical Science and Engineering, Konkuk University, Seoul, 05029 Republic of Korea

**Keywords:** Stem cells, Mechanisms of disease, Alzheimer's disease

## Abstract

Alzheimer’s disease (AD) is one of the progressive neurodegenerative diseases characterized by β-amyloid (Aβ) production and Phosphorylated-Tau (p-Tau) protein in the cerebral cortex. The precise mechanisms of the cause, responsible for disease pathology and progression, are not well understood because there are multiple risk factors associated with the disease. Viral infection is one of the risk factors for AD, and we demonstrated that Zika virus (ZIKV) infection in brain organoids could trigger AD pathological features, including Aβ and p-Tau expression. AD-related phenotypes in brain organoids were upregulated via endoplasmic reticulum (ER) stress and unfolded protein response (UPR) after ZIKV infection in brain organoids. Under persistent ER stress, activated-double stranded RNA-dependent protein kinase-like ER-resident (PERK) triggered the phosphorylation of Eukaryotic initiation factor 2 (eIF2α) and then BACE, and GSK3α/β related to AD. Furthermore, we demonstrated that pharmacological inhibitors of PERK attenuated Aβ and p-Tau in brain organoids after ZIKV infection.

## Introduction

Alzheimer’s disease (AD) is a progressive neurodegenerative disease that causes cognitive impairment and memory loss, characterized by β-amyloid (Aβ) and Phosphorylated-Tau protein (p-Tau) in the cerebral cortex [1, 2]. Although AD is prevalent dementia that is estimated to affect more than 50 million people worldwide, there are no efficient therapeutic drugs for patients. The detailed mechanisms responsible for AD pathology and progression are not well understood because there are multiple risk factors, including genetics, age, and environment, that could increase the risk of AD development [3]. Growing evidence among environmental risk factors includes infection, subsequent cellular stress, or neuroinflammation, as triggers for AD [4]. For example, neurotrophic viruses including Zika virus (ZIKV), Japanese encephalitis virus (JEV), herpes simplex virus (HSV), and cytomegalovirus (CMV) are capable of infecting neurons which could damage the central nervous system and ultimately lead to AD [5–8]. Cognitive impairment is also increasingly being reported in patients exposed to severe acute respiratory syndrome coronavirus 2 (SARS-CoV-2), a recently prevalent virus infection [9, 10]. The precise interaction between viral infections associated with the risk of developing AD has long been a subject of interest, however, direct causative effects could not be proven. Recently, proteomic analysis has shown that ZIKV infection promotes the differential expression of proteins linked to AD [6, 11]. Although many reports have indicated the possibility that AD pathology is related to ZIKV infection [12, 13], the molecular mechanisms of ZIKV underlying AD are still poorly understood.

ZIKV proteins are predominantly distributed near the endoplasmic reticulum (ER) and replicate in the ER of host cells [14, 15]. ZIKV induces massive vacuolization and perturbs ER-related pathways that are associated with apoptosis [16, 17]. Upregulated ER stress and the unfolded protein response (UPR) have been detected in the ZIKV-infected region of mouse embryos and human NSCs [15, 18]. Three major pathways involved in ER stress are initiated under altered ER homeostasis, including double-stranded RNA-dependent protein kinases (PKR)-like ER-resident kinase (PERK), inositol requiring 1 (IRE1), and activation transcription factor 6 (ATF6) [19–21]. The hyperactivated PERK pathway induced by persistent ER stress leads to hyperphosphorylation of downstream target Eukaryotic initiation factor 2 (eIF2α) and attenuates general protein synthesis, resulting in neurodegeneration [22–25]. In addition, levels of p-PERK and downstream p-eIF2 were found to be elevated in the cortex and hippocampus of postmortem AD brain, suggesting the association of the PERK-eIF2α pathway in AD pathogenesis [26–28]. In current studies, PERK, an upstream regulator in Aβ production and Tau phosphorylation, is highly involved in the neuroprotective role through the inhibition of PERK-eIF2α [27, 29–31]. Therefore, it is important to understand the involvement of ER stress in AD pathogenesis and the relevant signaling pathways for developing a promising therapeutic intervention. Indeed, it has been reported that ER stress was also increased in the recent epidemic SARS-CoV-2, and viral proteins are involved in the activation of ER stress transducers, including PERK and its downstream signals [32, 33].

Many researchers have employed brain organoids derived from human induced pluripotent stem cells (hiPSCs) to better understand human neural development and detailed pathology related to many neurodegenerative diseases [34–36]. Brain organoids resemble features of various brain regions and provide an opportunity to study brain development and understand detailed mechanisms of neurodegenerative diseases. To identify the molecular causes of AD-like phenotypes after ZIKV infection, we used 3D in vitro brain organoid systems to understand the mechanisms of AD development.

In this study, we hypothesized that persistent ER stress by ZIKV infection triggers the PERK-eIF2α pathway, resulting in elevations of AD pathology. This is the first report to study ZIKV-induced AD using a 3D-brain organoid model that encompasses aspects of the physiological disease, including Aβ plaque formation and p-Tau. This model will further elucidate the mechanism of ZIKV-mediated AD pathogenesis and allow future studies to identify potential downstream targets to treat this devastating disease.

## Results

### The characterization of brain organoids generated from AD patient-derived iPSCs

**Fig. 1:**
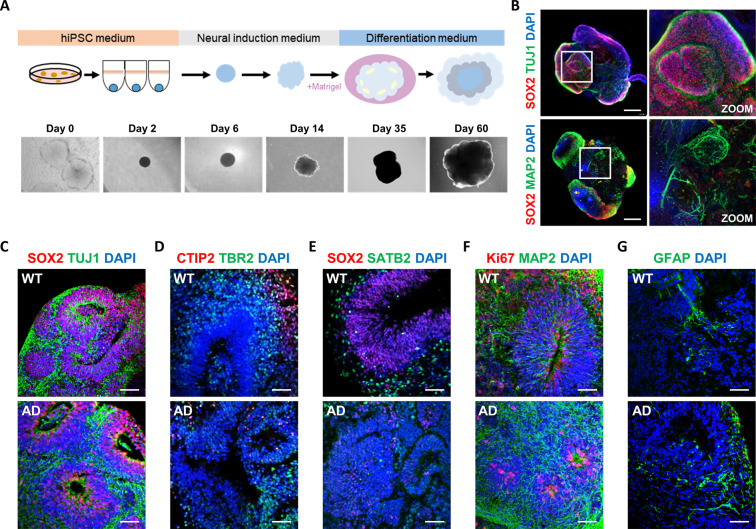
Generation and characterization of WT and AD hiPSCs-derived brain organoids. **A** A schematic procedure for generating brain organoids from hiPSCs. Representative bright-field images at different stages. **B** Clearing of whole organoids immunostained for neural progenitor marker SOX2 and the neuronal marker TUJ1 in WT and AD brain organoids at day 30. Scale bars, 500 µm. **C** Immunostaining of the neural progenitor marker SOX2 and the neuronal marker TUJ1 in WT and AD organoids at day 60. Scale bars, 100 µm. **D** Immunostaining of the cortical deep-layer marker CTIP2 and the intermediate progenitor marker TBR2 in WT and AD organoids at day 60. Scale bars, 100 µm. **E** Immunostaining of the neural progenitor marker SOX2 and the cortical upper layer marker SATB2 in WT and AD organoids at day 60. Scale bars, 100 µm. **F** Immunostaining of the proliferation marker Ki67 and the neuronal marker MAP2 in WT and AD organoids at day 60. Scale bars, 100 µm. **G** Immunostaining of the astrocyte marker GFAP in WT and AD organoids at day 60. Scale bars, 100 µm. Every experiment was performed in triplicate.

In order to model AD using human cells, we generated cerebral brain organoid models using wild-type (WT) and AD patient-derived iPSCs following the protocol described by Lancaster et al. [37]. Briefly, dissociated iPSCs were aggregated in 96-well plates for 5 days to generate embryoid bodies (EBs), which were transferred to 24-well plates for neural induction. After 6 days in a neural induction medium, the early organoids were coated in Matrigel and allowed to grow for 4 days. The organoids were moved and cultured in an orbital shaker to improve differentiation for at least 40 days (Fig. 1A). The organoids measured 2–3 mm in diameter and exhibited mature structures starting on day 60. The analysis of the brain organoids using the described clearing process and 3D imaging revealed a self-organized 3D structure (Fig. 1B). Whole-brain organoids were stained with SOX2 (neural progenitor marker) and TUJ1 (neuron). Different brain regions contain different cell populations, including progenitors and neurons migrating away from the ventricular zone. On day 60, sections of WT and AD brain organoids revealed neural progenitors positive for SOX2 in the ventricular zone and the expansion of TUJ1 neurons, forming a cortical layer (Fig. 1C). Because the cortex is one of the predominantly affected regions in AD patient’s brain, we characterized several markers related to the cortex layer in brain organoids. Both brain organoids showed a TBR2-positive subventricular zone-like layer and CTIP2-positive deep cortical layer on day 60 (Fig. 1D). The brain organoids included SATB2-positive cortical upper layer marker (Fig. 1E), MAP2-positive neurons, and Ki67-positive ventricles (Fig. 1F). Moreover, we also detected GFAP-positive astrocytes in both organoids at day 60 (Fig. 1G). Together, both brain organoids were derived from WT and AD iPSC displayed cortex-like structures containing AD pathology-associated cell types, such as neurons and astrocytes.

### Brain organoids from AD patient-derived iPSCs recapitulate AD pathology

**Fig. 2:**
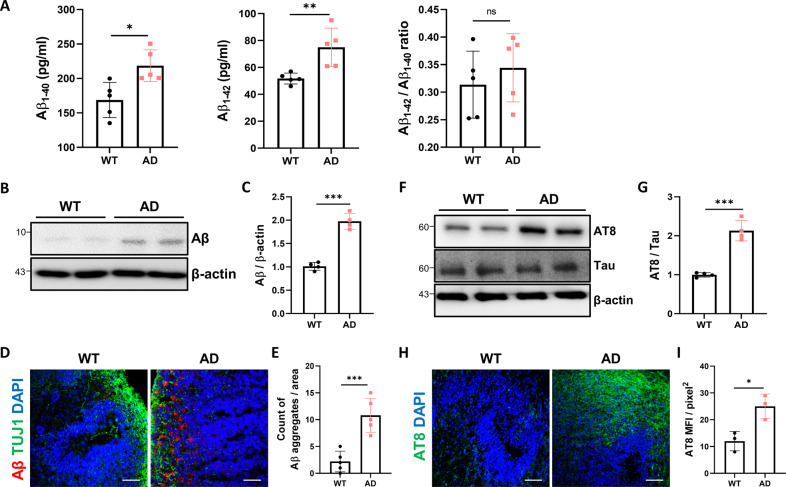
AD brain organoids exhibit AD-like phenotypes at day 60. **A** Lysates of WT organoids and AD organoids were analyzed by ELISA at day 60. The amounts of Aβ1-40 and Aβ1-42 in the RIPA fraction were measured by ELISA (*n* = 5). **B**, **C** Amounts of beta-amyloid were assessed by western blotting analysis in WT and AD organoids at day 60 (*n* = 4). **D** Representative images of the immunostaining for Aβ and TUJ1 in WT and AD organoids at day 60. **E** Quantification of Aβ immunoreactivity and particle counts in WT and AD organoids (*n* = 5). Scale bars, 50 µm. Aβ levels detected by D54D2 antibody. **F**, **G** Total tau and p-tau levels of WT and AD organoids were analyzed by western blotting and quantified. **H**, **I** Representative images of immunostaining for p-Tau in WT and AD organoids at day 60 (*n* = 3). The p-tau levels detected by AT8 (Ser202/Thr205) antibody (*n* = 3). Scale bars, 50 µm. n.s.: not significant, *P < 0.05, **P < 0.01, and ***P < 0.001. The data are presented as the mean ± SD.

AD is characterized by the accumulation of extracellular Aβ plaques and intracellular p-Tau. To investigate whether the major AD pathologies were recapitulated in AD brain organoids, an increase in Aβ level was confirmed by Aβ ELISA (Fig. 2A). Organoids were lysed with RIPA buffer, and Aβ40 and Aβ42 levels were measured on day 60. In agreement with the previous study, we detected significantly higher levels of Aβ40 and Aβ42 from AD organoids compared to WT organoids. Consistent with the results, western blotting with Aβ antibodies also revealed higher levels of Aβ in AD organoids compared to WT organoids (Fig. 2B, C). We also stained brain organoids with Aβ antibodies at day 60 and found more Aβ aggregation in AD organoids compared to WT organoids (Fig. 2D, E). Next, we assessed the level of p-Tau in brain organoids using AT8 antibody that recognizes phosphorylated Ser202 and Thr205 of Tau protein (Fig. 2F, G). The expression level of p-Tau was higher in AD organoids compared to WT organoids. Consistent with the western blotting, the expression of immunostaining with AT8 was higher in AD organoids compared to WT organoids at day 60 (Fig. 2H, I). These brain organoids are suitable for recapitulating AD-like pathologies, including upregulated Aβ and p-Tau.

### ZIKV infection induces Aβ accumulation through increasing BACE abundance

**Fig. 3:**
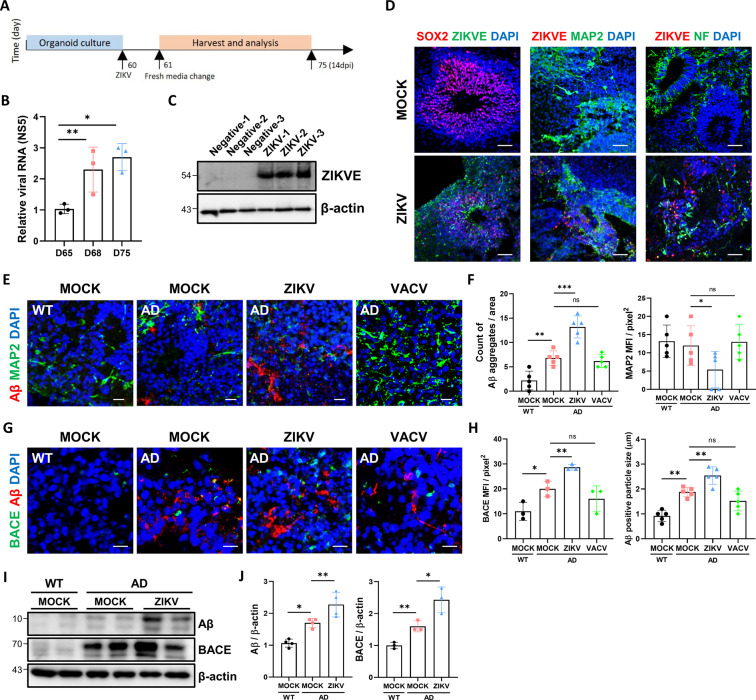
ZIKV infection induces Aβ accumulation through increasing BACE abundance. **A** Schematic of the Zika virus (ZIKV) infection procedure. Organoids were infected on day 60 and analyzed 14 dpi. **B** RT-qPCR analysis of the NS5 gene in ZIKV-infected organoids at 4 dpi (Day 65), 7 dpi (Day 68) and 14 dpi (Day 75). **C** ZIKV envelope (ZIKVE) protein level of each organoid 14 dpi. **D** Immunostaining for ZIKVE, SOX2, MAP2, and Neurofilament (NF) markers in organoids exposed to ZIKV 14 dpi. Scale bars, 50 µm. **E** Immunostaining of Aβ and MAP2 (neuron marker) in WT and AD organoids exposed to mock conditions, ZIKV and vaccinia virus (VACV). Scale bars, 10 µm. **F** Counts of Aβ aggregates per area and quantification of MAP2 expression (*n* = 5). **G** Immunostaining of Aβ and BACE in WT and AD organoids exposed to mock conditions, ZIKV and VACV. Scale bars, 10 µm. **H** Quantification of Aβ positive particle size (*n* = 5) and BACE positive cells (*n* = 3). **I** Western blotting of Aβ, BACE and β-actin in WT and AD organoids exposed to mock conditions and ZIKV. **J** Quantification of Aβ (*n* = 4) and BACE (*n* = 3) normalized to β-actin. n.s.: not significant, *P < 0.05, **P < 0.01, and ***P < 0.001. The data are presented as the mean ± SD.

We hypothesized that ZIKV infection can trigger AD pathologies such as Aβ deposition and elevated p-Tau level in the brain. We infected 60-day-old organoids and cultured them for an additional 14 days prior to analysis. The 60-day-old brain organoids were infected with ZIKV for 24 h, and the medium was replaced with a differentiation medium (Fig. 3A). The abundance of infectious virions in the supernatants of organoid culture medium after infection was determined by plaque assay, which confirmed the time dependence of the virus replication (Fig. S1A–B). The formation of plaques by ZIKV was continuous until 6 days post infection (dpi), but it slightly decreased after 14 dpi. Concomitantly, ZIKV-infected organoids were subjected to RT-PCR with primers specific for ZIKV NS5 4 dpi, 7 dpi, and 14 dpi (Fig. 3B). The level of ZIKV NS5 RNA expression was increased constantly over time, representing a productive infection. ZIKV envelope (ZIKVE) protein was also detected by western blotting in organoids after ZIKV infection (Fig. 3C). Staining of the ZIKVE and neural progenitor cell marker SOX2 showed that these proteins were expressed in brain organoids 14 days after infection. Furthermore, the protein levels of neurofilament and MAP2 were decreased in ZIKV infected organoids (Fig. 3D). To determine whether ZIKV infection accelerates AD-related phenotypes, first, we analyzed Aβ pathology in organoids after ZIKV infection. Accelerating increased levels of Aβ was confirmed by immunostaining in ZIKV-infected AD brain organoids (Fig. 3E, F). We also examined the effects of ZIKV compared with those of vaccinia virus (VACV) infection on brain organoids to determine whether Aβ pathology is a specific phenotype of ZIKV infection. The formation of plaques by VACV was continuous until 6 dpi, but it slightly decreased after 14 dpi (Fig. S1C–D). In addition, we examined the effects of ZIKV compared with those of VACV infection on brain organoids to determine whether a neurogenic defect is also a specific phenotype of ZIKV infection. Although mock and VACA-treated AD organoids grew over time, the size of ZIKV-infected brain organoids was downregulated (Fig. S2A–B). The expression level of neurofilament (NF) and MAP2 were downregulated in ZIKV infected organoids, but there was no decrease in VACV infected organoids (Fig. S2C–F). Next, we investigated the mechanisms underlying ZIKV infection-induced Aβ production. Aβ is processed from the amyloid precursor protein (APP) through sequential cleavages, first producing a beta C-terminal fragment by BACE and then Aβ by γ-secretase complex [38]. In the amyloidogenic pathway, BACE, which is a critical enzyme that drives Aβ production is increased in AD patients compared to WT [39]. It is hypothesized that ZIKV infection could accelerate Aβ aggregation by increasing BACE expression. Indeed, the abundance of BACE protein and Aβ was significantly increased in ZIKV-infected AD brain organoids, compared to non-infected AD and VACV-infected organoids (Fig. 3G, H). Moreover, increased expression of BACE and Aβ levels was observed by western blotting in ZIKV-infected AD organoids compared to non-infected organoids (Fig. 3I, J). Taken together, ZIKV infection accelerated AD pathology and elevated BACE expression as one of the major mechanisms underlying Aβ processing.

### ZIKV infection enhances p-TAU levels in AD organoids through GSK3α/β

**Fig. 4:**
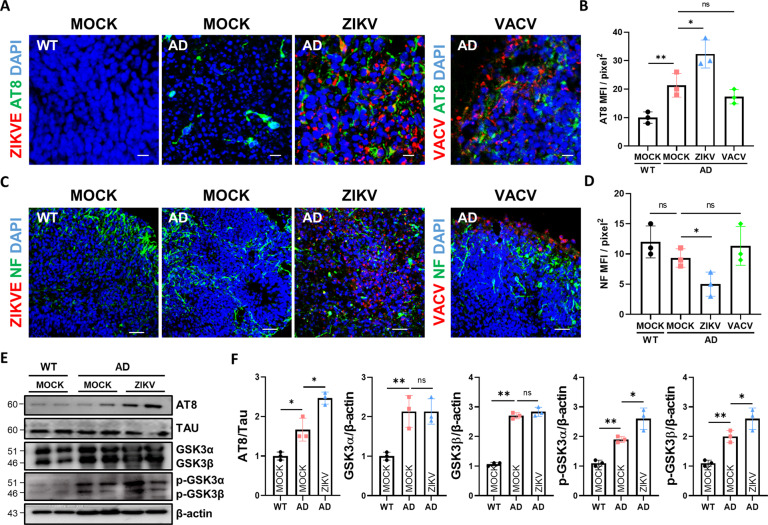
ZIKV infection enhances p-TAU levels in AD organoids through GSK3α/β. **A** Representative images of immunostained p-tau with AT8 (Ser202/Thr205), ZIKVE, and VACV antibodies. Scale bars, 10 µm. **B** Each graph bar represents the percentage of AT8-positive cells in the organoids. (*n* = 3). **C** Representative images of immunostained NF and ZIKVE antibodies. Scale bars, 50 µm. **D** Each graph bar represents the percentage of NF-positive cells in the organoids (*n* = 3). **E** Western blotting of p-Tau (AT8), Tau, p-GSK3α/β, GSKα/β and β-actin in WT and AD organoids exposed to mock conditions and ZIKV. **F** Quantification of p-Tau (AT8), Tau, p-GSK3α/β, GSKα and GSK3β levels. p-Tau was normalized to total Tau. The others were normalized to β-actin (*n* = 3). n.s.: not significant, *P < 0.05, and **P < 0.01. The data are presented as the mean ± SD.

Next, we also examined the level of p-Tau, one of the other major pathologies of AD, in AD organoids after ZIKV infection. ZIKV infection significantly increased p-Tau level in ZIKV-infected AD brain organoids compared to AD brain organoids exposed to mock conditions (Fig. 4A, B). We also examined the effects of ZIKV compared with those of VACV infection on brain organoids to determine whether p-Tau pathology is a specific phenotype of ZIKV infection. The level of p-Tau in AD organoids exposed to VACV didn’t show any increase from those of AD organoids exposed to mock-conditions (Fig. 4A, B). Neurofilament proteins are components of neurofibrillary tangles, and NF as well as other microtubule proteins are impaired in AD. The immunofluorescence intensity of NF was downregulated in ZIKV-infected AD organoids compared to WT or AD organoids. In addition, VACV infection in AD organoids had no effect on NF homeostasis (Fig. 4C, D). We then investigated the underlying mechanisms of elevated p-Tau levels due to ZIKV infection. Phosphorylated-Tau protein is processed by several kinases, including glycogen synthase kinase 3 (GSK3), cyclin-dependent protein kinase 5 (Cdk5), mitogen-activated protein kinase (MAPK) [40]. Above all, GSK3 is a ubiquitously expressed serine/threonine kinase that plays a key role in tau aggregation of AD pathology [40]. The basal levels of both p-Tau and p-GSK3α/β (Y216/Y279) was upregulated in AD organoids compared with WT organoids (Fig. 4E, F). We also found that both p-GSK3α/β and total GSK3α/β were increased substantially in ZIKV-infected brain organoids through western blotting (Fig. 4E, F). Together, ZIKV infection showed the pathology of accelerated p-Tau and elevated GSK3α/β expression as one of the underlying mechanisms.

### PERK-eIF2α activation is associated with AD phenotypes in ZIKV-infected organoids

**Fig. 5:**
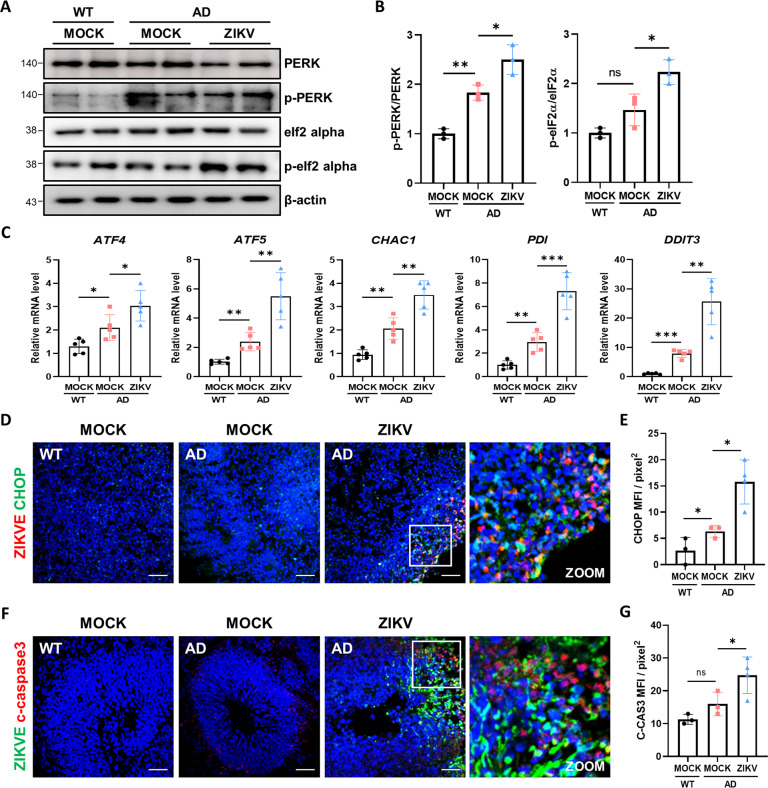
PERK-eIF2α activation is associated with AD phenotypes in ZIKV-infected organoids. **A** Western blotting of PERK, p-PERK, eIf2α, p-eIf2α, and β-actin in WT and AD organoids exposed to Mock conditions and ZIKV. **B** Quantification of p-PERK and p-eIf2α. p-PERK was normalized to total PERK and p-eIf2α was normalized to total eIf2α (*n* = 3). **C** RT-qPCR analysis of *ATF4, ATF5, CHAC1, PDI*, and *DDIT3* levels in total RNA extracts obtained from ZIKV-infected AD brain organoids and mock-treated samples on day 75 (*n* = 5). **D** Immunostaining of WT and AD organoids exposed to mock and ZIKV showing ZIKVE and CHOP (ER stress marker). White square shows ZIKV-infected area. Scale bars, 50 µm. **E** Quantification of CHOP-positive cells 14 dpi (*n* = 3). **F** Immunostaining of WT and AD organoids exposed to mock and ZIKV showing ZIKVE and cleaved caspase3 (apoptosis marker). White square shows ZIKV-infected area. Scale bars, 50 µm. **G** Quantification of cleaved caspase3-positive cells 14 dpi (*n* = 3). n.s.: not significant, *P < 0.05, and **P < 0.01. The data are presented as the mean ± SD.

ER stress can be activated by several signaling pathways, including viral infection, calcium regulation, and glucose deprivation [41, 42]. Dysfunction caused by ER stress coupled to the accumulation of misfolded or unfolded proteins and activation of cell death pathways also contributes to neurodegenerative disorders, including AD [43]. To investigate whether PERK and eIF2α were activated after ZIKV infection in brain organoids, the protein level of both activated PERK and eIF2α was examined by western blotting. Levels of p-PERK and p-eIF2α were higher in AD organoids compared to WT organoids, and the increase was greater in AD organoids exposed to ZIKV (Fig. 5A, B). To further investigate whether PERK and eIF2α were activated after ZIKV infection in brain organoids, we used quantitative real-time PCR to analyze several genes related to ER stress and UPR pathways using RNA extracted from brain organoids on day 38 (Fig. 5C). The PERK branch of UPR-associated genes, including *ATF4, ATF5 CHAC1, PDI*, and *DDIT3*, were upregulated upon infection. In addition, ER stress acceleration was analyzed by western blotting, with the upregulation of ER stress marker, clanexin and protein disulfide isomerase (PDI) in ZIKV-infected AD organoids (Fig. S3A–B). We also detected increased immunofluorescence staining for the ER stress marker calnexin in AD organoids exposed to ZIKV compared to mock conditions (Fig. S3C–D). When UPR-induced factors fail to alleviate ER stress, the extrinsic and intrinsic apoptotic pathways are activated. Persistent ER stress in infected organoids subsequently triggers terminal UPR and apoptosis through upregulation of CHOP [44–46]. To examine the terminal UPR and apoptosis via upregulation of CHOP, we stained brain organoids with anti-CHOP and found more CHOP expression in ZIKV-infected brain organoids compared to mock conditions (Fig. 5D, E). In addition, cleaved caspase3 was also analyzed by immunostaining to evaluate the apoptotic effect of ZIKV in brain organoids (Fig. 5F, G). The results indicate that ZIKV infection induces and aggravates ER stress and that cellular signaling is eventually associated with cell death in AD pathologies.

### PERK inhibition attenuated AD phenotypes including Aβ and p-Tau

**Fig. 6:**
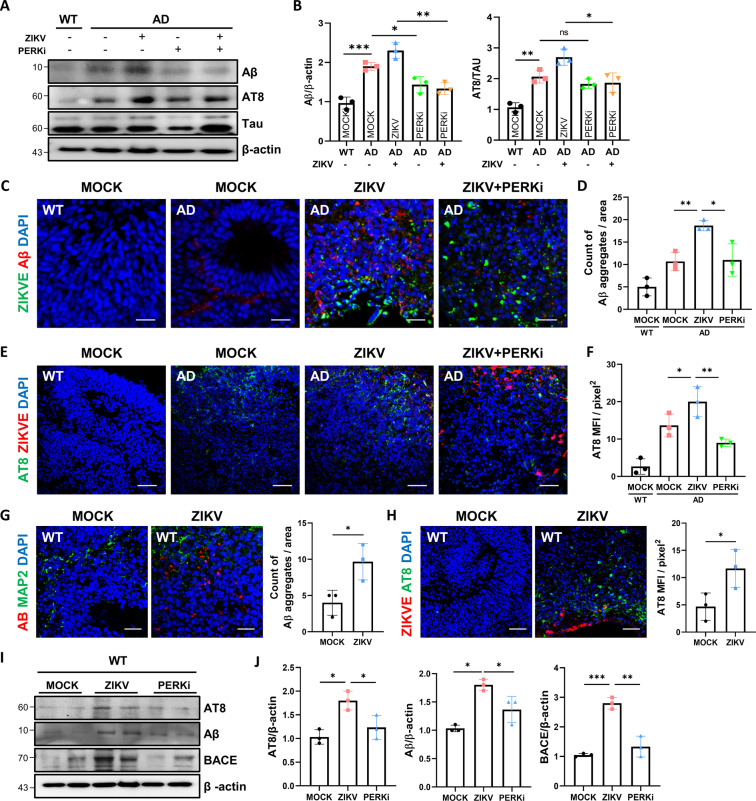
PERK inhibitor attenuated AD phenotypes including Aβ and p-Tau. **A** PERK inhibitor (GSK2656157) was administrated for two weeks after ZIKV infection. Representative western blotting results showing Aβ, p-Tau, Tau, and β-actin. **B** The quantification of Aβ and p-Tau. Aβ was normalized to β-actin and p-Tau was normalized to Tau protein (*n* = 3). **C** Representative immunofluorescence image showing Aβ aggregation in each organoid. Scale bars, 50 µm. **D** The level of Aβ was analyzed in each organoid 14 dpi. The bar graph shows the quantification of Aβ aggregates per area in WT and AD organoids exposed to mock conditions, ZIKV and PERKi treatment after ZIKV infection (*n* = 3). **E** Representative immunofluorescence image showing p-Tau with AT8 (Ser202/Thr205) in each organoid. Scale bars, 50 µm. **F** The level of p-Tau was analyzed in each organoid 14 dpi. The bar graph shows the quantification of p-Tau immunoreactivity in AD organoids exposed to mock conditions, ZIKV, and PERKi treatment after ZIKV infection (*n* = 3). **G** Representative immunofluorescence images showing Aβ and MAP2 in WT organoids exposed to mock conditions and ZIKV (*n* = 3). Scale bars, 50 µm. **H** Representative immunofluorescence images showing p-Tau and ZIKVE in WT organoids exposed to mock conditions and ZIKV (*n* = 3). Scale bars, 50 µm. **I** Western blotting showing Aβ, BACE, p-Tau, and β-actin in WT organoids. **J** Quantification of Aβ and p-Tau was normalized to β-actin (*n* = 3). n.s.: not significant, *P < 0.05, and **P < 0.01. The data are presented as the mean ± SD.

To assess whether ZIKV directly affects AD pathology through PERK-eIF2α activation, GSK2656157 (PERK inhibitor, PERKi), an inhibitor of the upstream factor, was treated in ZIKV-infected organoids. First, the level of Aβ was downregulated by PERKi treatment in AD organoids, suggesting that Aβ production was due to PERK activation (Fig. 6A, B). However, the p-Tau level did not show a significant difference between AD and PERKi-treated AD organoids. Next, we compared the level of AD pathologies in ZIKV-infected organoids and AD organoids administrated with ZIKV + PERKi. Surprisingly, PERKi treatment downregulated both Aβ and p-Tau expression in ZIKV-infected AD organoids (Fig. 6A, B). Consistently, immunostaining with anti-Aβ also revealed lower expression levels in AD organoids administrated with ZIKV + PERKi compared to ZIKV-infected organoids (Fig. 6C, D). The p-Tau expression was also significantly decreased in AD organoids administrated with ZIKV + PERKi compared to ZIKV-infected AD organoids (Fig. 6E, F). These results represent that the acceleration of Aβ production and Tau phosphorylation is increasing via PERK-eIF2α arm. To investigate whether the ZIKV infection can also trigger AD pathologies in WT organoids, we infected ZIKV in WT organoids and analyzed them in the same manner as we examined in AD organoids. The level of Aβ production in ZIKV-infected WT organoids was increased compared to mock (Fig. 6G). In addition, the level of p-Tau was increased in ZIKV-infected organoids compared to mock (Fig. 6H). To investigate whether the increase in p-Tau level was triggered by PERK-eIF2 in WT organoids, we also treated PERKi in ZIKV-infected organoids. Western blotting also showed lower levels of Aβ and p-Tau in organoids administrated with ZIKV + PERKi compared to ZIKV-infected organoids (Fig. 6I, J). In conclusion, this study demonstrated that the predominant mechanism of ZIKV-induced AD pathologies is PERK-eIF2α regulating the AD-associated factors, including GSK3 and BACE, and that targeting PERK activation by inhibitors has potential therapeutic implications in AD pathologies.

## Discussion

There are many risk factors to cause and damage the brain responsible for AD progression. Recent investigations suggest that AD pathologies may also be triggered in the brain through one of the risk factors such as neurotrophic virus infections [47–49]. Here, we generated a human brain organoid model to investigate the underlying mechanisms of ZIKV infection responsible for AD pathologies, including accelerated Aβ production and p-Tau. The levels of Aβ production and p-Tau were accelerated in AD organoids exposed to ZIKV as well as WT organoids exposed to ZIKV.

ZIKV infection leads to the accumulation of viral proteins in the ER and sustained ER stress and UPR activation [17, 42]. It has been demonstrated that the PERK-related UPR pathway is activated in the ZIKV-infected mouse model [15]. Activated ER stress is also a risk factor in neurodegenerative diseases, including AD, Parkinson’s disease, and other disorders [50–52]. Three ER transmembrane receptors, PERK, ATF6, and IRE1, play roles as stress sensors and mediators of downstream signals [53, 54]. During ER stress, each of the three receptors is activated by phosphorylation (PERK and IRE1) or cleavage (ATF6) to maintain ER homeostasis. Several studies have reported that PERK/eIF2α pathway is detected in the mouse AD model and postmortem AD patient’s brain [55–57]. Overactivation of PERK triggers phosphorylation of downstream eIF2α, leading to synaptic failure, neuronal impairment, and cellular death. It is also documented that PERK-eIF2α pathway contributes to AD pathologies as an upstream regulator. Aβ production is triggered through BACE1 abundance and Tau phosphorylation is upregulated through GSK3α/β affected by PERK-eIF2α pathway [39, 55, 58]. In addition, it is also reported that PERK inhibitor treatment in AD mouse model, such as 5XFAD or APP/PS1 significantly decreases phosphorylated PERK and eIF2α [26, 27, 59].

Our data also demonstrated that both ER stress and UPR activation are activated in ZIKV-infected brain organoids and accompanied the upregulation of AD pathologies, including Aβ and p-Tau. It is proved that AD pathologies proceeded through the same axis, upregulation of BACE1 and GSK3α/β from PERK-eIF2α pathway. Furthermore, direct inhibition of PERK (GSK2656157) in ZIKV-infected organoids showed restored AD pathologies and UPR-mediated apoptosis. These findings that the PERK inhibition could be a promising therapeutic target for drug research for AD in which UPR pathway is implicated. In addition, PERKi treatment was effective to rescue the neurogenic effect in organoids exposed to ZIKV infection (Fig. S4A–B). In these reasons, ER stress activated by ZIKV have a causal relationship to AD progression through PERK/eIF2α.

Recent 3D brain organoid models that closely recapitulate the human brain are useful for disease modeling, drug screening, and deep understanding of human brain development [34]. In this study, we generated both cerebral brain organoids derived from WT and AD patients iPSCs and applied ZIKV infection to brain organoids to study the mechanisms underlying ZIKV-induced AD pathology. Although cerebral brain organoids are a physiologically potential application model for the human brain, there are challenges and limitations for improvement [60, 61]. For instance, the absence of blood vessels or immune cells is required to investigate blood-brain barrier leakage and immune responses from brain pathogens [62]. Further studies are needed to identify other critical events after virus infection cytokine storm stimulation or blood-brain barrier leakage using advanced brain organoids.

Taken together, this study first reports ZIKV contributes to AD pathology via the PERK/eIF2α pathway, accelerating Aβ production and p-Tau in brain organoids. We conclude that targeting the activated-ER stress by inhibitors is a potential therapeutic strategy aimed at lowering the incidence of ZIKV-associated AD phenotypes in the brain.

## Materials and methods

### Zika virus production and titration

Asia strain ZIKV FLR (Accession: KU820897) and vaccinia virus western reserve strain (Accession: AY243312) were obtained from ATCC (VA, USA). Every infectious ZIKV was conducted under biosafety level 2 (BSL 2) conditions at Konkuk University following approved institutional biosafety guidelines. All ZIKV stocks were propagated in Vero cells, and virus plaque assays were performed on 2D cultures of Vero cells in six-well plates. VACV was propagated in HeLa cells, and the titer was measured by plaque assay in 143 TK-cells. For the confirmation of the infectious virus in brain organoids, for each indicated time point in the brain organoid culture, the medium was diluted and evaluated by plaque assay.

### Maintenance of human iPSCs

We obtained the KSCBi005-A human-induced pluripotent stem cell line from the National Stem Cell Bank of Korea (KSCB) for the generation of wild-type brain organoids. These iPSCs were maintained in Essential 8 media (Gibco) on dishes precoated with vitronectin (Gibco). The cells were passaged every 4–5 days using ReLeSR™ reagent (STEMCELL Technologies). For the generation of AD brain organoids, we obtained patient-derived iPSC CS40iFAD-nxx cells, which have a PSEN1 mutation, from Cedar-Sinai. These cells were grown in mTESR plus medium (Gibco) on dishes precoated with GFR-matrigel. The cells were passaged every 5–6 days using ReLeSR™.

### Generation of brain organoids

We followed the brain organoid protocols that were previously described by Lancaster et al. [37] Briefly, on day 0 of organoid culture, iPSCs were dissociated into single cells by TrypLE™ express enzyme (Thermo Fisher). The dissociated single cells were transferred to an ultralow binding 96-well plate (Corning), and 9000 cells were plated in each well with human ES medium with 4 ng/ml basic fibroblast growth factor (Sigma-Aldrich) and 50 µM Rho-associated protein kinase inhibitor. On day 3, the medium was replaced with fresh medium. When the size of the EBs was greater than 500 µm, the EBs were transferred to low-adhesion 24-well plates and seeded in neural induction medium containing DMEM-F12 supplemented with 1X N2 supplement (Gibco), 1 µg/ml heparin solution, 1X Gluta-MAX (Gibco), and 1X MEM-NEAA (Gibco). After 6 days of culture in neural induction media, neuroepithelial tissues were transferred to droplets of Matrigel (Corning). These droplets were incubated at 37 °C for 20 min and subsequently detached from the Parafilm and cultured in cerebral organoid differentiation medium (CODM) containing a 1:1 mixture of DMEM/F12: Neurobasal medium supplemented with 1X N2 supplement (Gibco), 1X B27 supplement without vitamin A (Gibco), 2-mercaptoethanol, 1X insulin (Sigma), 1X GlutaMAX (Gibco) and 1X MEM-NEAA (Gibco). After four days of stationary growth, the medium was changed, and the organoids in CODM with 1X N2 supplement (Gibco), 1X B27 supplement with vitamin A (Gibco), 2-mercaptoethanol, 1X insulin (Sigma), 1X GlutaMAX (Gibco) and 1X MEM-NEAA (Gibco) were transferred to an orbital shaker.

### Infection of the brain organoids

On day 60, the brain organoids were infected with ZIKV diluted in CODM at 0.2 MOI and 2 MOI. The cells were subjected to ZIKV exposure for 24 h, and then, the medium was replaced with virus-free CODM and incubated at 37 °C in a 5% CO_2_ incubator. Brain organoids were maintained in cerebral organoid differentiation medium with the medium changed every other day.

### Inhibitor treatment

For PERK inhibition, brain organoids were treated with 10 µM GSK2656157 (Cayman) after ZIKV infection. Brain organoids were infected with the Asian ZIKV strain at a MOI of 0.2. After 24 h, the virus-containing medium was replaced with fresh media, and inhibitors were added. The effect of PERKi on the treated organoids was analyzed 14 dpi in the same way as the mock-treated organoids were analyzed.

### RNA extraction and qRT-PCR analysis

For each time point, at least 10 organoids were washed with PBS and lysed in TRIzol (Invitrogen) for total RNA extraction following the manufacturer’s instructions. Complementary DNA synthesis was performed using a Superscript III First-Strand kit (Invitrogen) according to the manufacturer’s instructions. qRT-PCRs were performed with SYBR Green PCR Mix (Applied Biosystems) on a 7500 Real-Time PCR system. Each sample was analyzed after normalization to the level of the housekeeping gene, GAPDH. The following primers were used for qRT-PCR. GAPDH, forward 5'-TGA TGA CAT CAA GAA GGT GGT G-3' and reverse 5'-TGA TGA CAT CAA GAA GGT GGT G-3'; *ATF4*, forward 5′-CCA ACA ACA GCA AGG AGG AT-3′ and reverse 5′-AGG TCA TCT GGC ATG GTT TC-3′; *ATG5*, forward 5′-GCT CGT AGA CTA TGG GAA ACT CC-3′ and reverse 5′-CAG TCA TCC AAT CAG AGA AGC CG-3′; *DDIT3*, forward 5′-ATG GAG CTT GTT CCA GCC AC-3′ and reverse 5′-GTG TCC CGA AGG AGA AAG GC-3′; *CHAC1*, forward 5′-GTG TGG TGA CGC TCC TTG-3′ and reverse 5′-TGC TTA CCT GCT CCC CTT G-3′; and *PDI* forward 5′-CAAGATCAAGCCCCACCTGAT-3′ and reverse 5′-AGTTCGCCCCAACCAGT ACTT-3′.

### Immunostaining and imaging

For each time point and condition, organoids were fixed overnight with 4% paraformaldehyde at 4 °C for immunofluorescence staining. After washing with PBS more than three times, the organoids were pooled into 15-ml conical tubes with 30% sucrose solution and incubated overnight, then embedded in 10% gelatin + 30% sucrose solution and frozen in a deep-freeze. Organoids were cryo-sectioned into 15-µm thick slices. The organoids sections were washed in PBS and permeabilized in sodium citrate pH 6.0 at 95 °C for 5 min. After permeabilization, the samples were washed three times with PBS and blocked with 5% NGS blocking solution containing 0.1% Triton X-100. The sections were incubated overnight with primary antibodies and diluted 5% NGS blocking solution containing 0.2% Triton X-100 at 4 °C. After three washes with PBS, the sections were incubated with secondary antibodies at room temperature (RT) for one hour, and the nuclei were stained with DAPI (Zymed Laboratories Inc.) for 10 min at RT. Finally, the sections were washed in PBS and mounted with DAKO fluorescence mounting medium (Agilent Pathology Solutions). Information on the primary antibodies and secondary antibodies is listed in Supplementary Table 1. The cells were counted with ImageJ software for analysis of the immunostaining density to determine the expression level of proteins. For clearing organoids in preparation for 3D imaging analysis, the organoids were prepared with a Cyto Vista 3D cell culture clearing/staining kit (Invitrogen) following the manufacturer’s instruction.

### Western blotting

Organoids were harvested after PBS washing and resuspended organoids were lysed in 300 µl of Pro-prep protein lysis buffer (Intron Biotechnology). The protein samples were resolved by SDS-PAGE and transferred to nitrocellulose membranes. The membranes were blocked with a 3% bovine serum albumin (BSA) solution and then incubated with agitation overnight. The signals were detected with an enhanced chemiluminescence (ECL) detection kit (GE Healthcare Life Science). The primary antibodies used are listed in Supplementary Table 1.

### ELISA

The protein levels of Aß40 and Aß42 in RIPA buffer were measured using a human amyloid beta [40] ELISA kit (Thermo Fisher) and human amyloid beta [42] ELISA kit according to the manufacturer’s instructions. The total levels of samples were quantified by BCA analysis. Total Aß40 and Aß42 levels in the RIPA lysates of 5–6 organoids in each group were analyzed by ELISA.

### Statistical analysis

Data analyses were performed with Prism 9 software (GraphPad Software), and statistical tests were examined according to the variance, distribution, and normality of each dataset. Statistical analyses were performed using unpaired two-tailed Student’s *t*-tests for parametric datasets. Levels of significance: ***P < 0.001, **P < 0.01, and *P < 0.05 for all statistics herein; and ns indicates not significant. The number of biological replicates in each experiment is specified in the figure legends. Every sample was selected randomly for analysis.

## Supplementary information

Supplementary information

Original Data File

## Data Availability

All data generated or analyzed during the current study are available from the corresponding author on reasonable request.
