# Supplementary material for: Zika virus infection accelerates Alzheimer’s disease phenotypes in brain organoids
Source: Cell Death Discov. 2022 Apr 2;8:153. doi: 10.1038/s41420-022-00958-x (PMC8976422; doi:10.1038/s41420-022-00958-x)
Supplement: Supplementary file 1 — Supplementary information [file 41420_2022_958_MOESM1_ESM.docx]

**Supplementary figures**

**
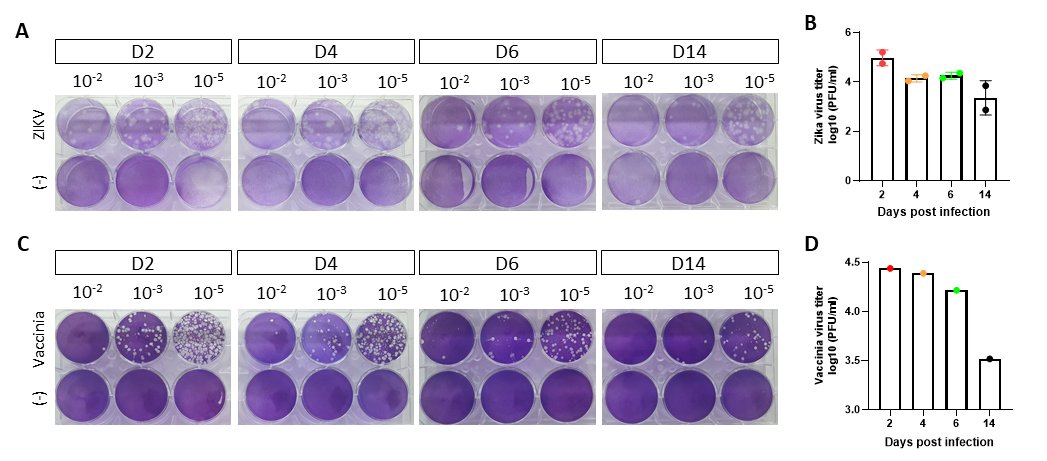
**

**Fig. S1. ZIKV and VACV replication in brain organoids.**

(**A**) Representative bright-field images of the plaque assay of ZIKV-infected organoids on days 2, 4, 6, and 14. The supernatant was obtained from brain organoids culture medium and serially diluted. (**B**) Quantification of the plaque-forming units of ZIKV on days 2, 4, 6, and 14. Log 10 PFU plotted against the serially diluted virus. (**C**) Representative bright-field images of the plaque assay of Vaccinia virus (VACV)-infected organoids on days 2, 4, 6, and 14. The supernatant was obtained from brain organoids culture medium and serially diluted. (**D**) Quantification of the plaque-forming unit of VACV on days 2, 4, 6, and 14. Log 10 PFU plotted against the serially diluted virus.

**
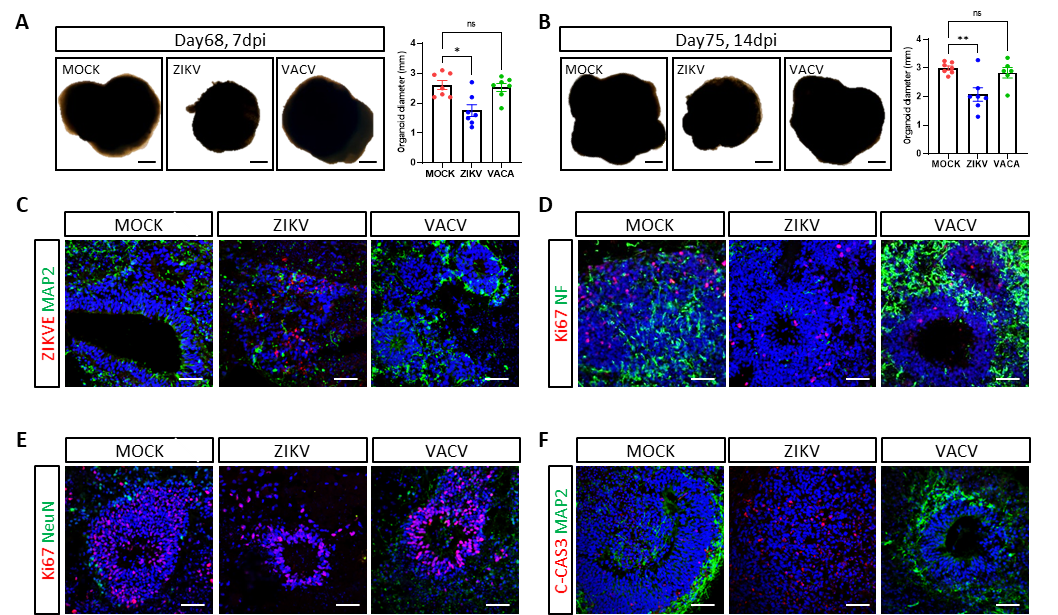
**

**Fig. S2. ZIKV infection impaired brain organoids compared to mock- and VACV-treated organoids.**

(A) Representative bright-field images obtained 7 dpi. Quantification of the diameter of the organoids exposed to ZIKV, VACV, and mock treatment (n=7). Scale bars, 500 µm. (B) Representative bright-field images obtained 14 dpi. Quantification of the diameter of the organoids exposed to ZIKV, VACV, and mock treatment (n=7). Scale bars, 500 µm. (C-F) Immunostained ZIKVE (Zika virus envelope), MAP2, NeuN (mature neuron marker), Ki67 (proliferation marker), and c-caspase3 (apoptosis marker) in organoids exposed to ZIKV and VACV 7 dpi. Scale bars, 50 µm. *P < 0.05, **P < 0.01, ***P < 0.001. Data are presented as the mean ±SD.


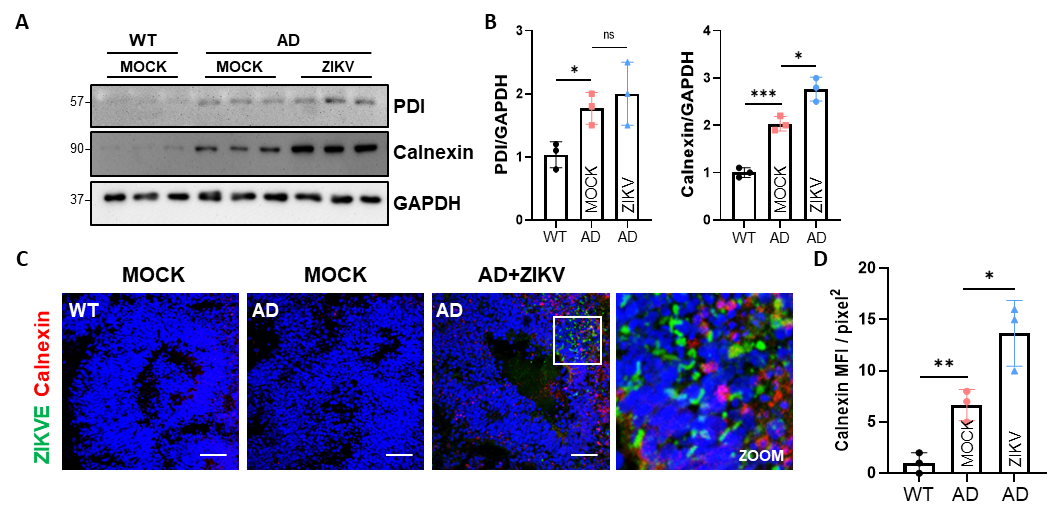


**Fig. S3. ER stress was activated in AD organoids after ZIKV infection.**

(A) Western blot analysis of PDI and calnexin (ER stress marker) was carried out using lysates of mock- and ZIKV-infected organoids 14 dpi. (B) Quantification of PDI and calnexin in three independent samples and normalized to β-actin (n=3). (C) Representative images of the immunostained calnexin and ZIKE, and (D) quantification in AD organoids exposed to mock conditions and ZIKV (n=3). *P < 0.05, **P < 0.01, ***P < 0.001. Data are presented as the mean ±SD


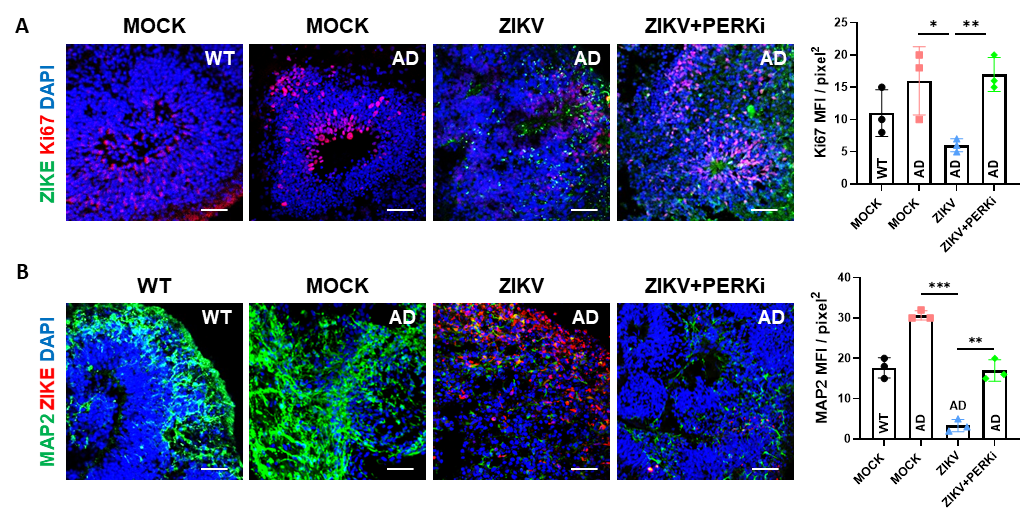


**Fig. S4. The effect of PERKi treatment in brain organoids.**

(A) Representative images of the immunostained Ki67 and ZIKE, and the quantification of Ki67 expression (n=3). (B) MAP2 and ZIKE expression in each organoid, and the quantification of MAP2 expression in each organoid (n=3). Scale bars, 50 µm. *P < 0.05, **P < 0.01, ***P < 0.001. Data are presented as the mean ±SD

**Supplementary Table**

Supplementary Table 1. List of antibodies used in this study.

| **Name** | **Species** | **Dilution** | **Vendor** | **Catalog #** |
| --- | --- | --- | --- | --- |
| Zika virus Envelope | Rabbit | 1:2000 | GeneTex | GTX133314 |
| Zika virus Envelope | Mouse | 1:1000 | GeneTex | GTX634155 |
| Vaccinia virus | Rabbit | 1:1000 | GeneTex | GTX36578 |
| SOX2 | Rabbit | 1:1000 | Abcam | ab5603 |
| TUJ1 | Mouse | 1:1000 | Biolegend | 801202 |
| MAP2 | Mouse | 1:1000 | Sigma | MAB3418 |
| Ki67 | Rabbit | 1:500 | Abcam | ab15580 |
| Neurofilament | Mouse | 1:500 | Cell signaling | 2836s |
| CTIP2 | Rat | 1:500 | Abcam | ab18465 |
| SATB2 | Mouse | 1:500 | Abcam | ab34735 |
| GFAP | Mouse | 1:500 | Cell signaling | 3670S |
| c-caspase3 | Rabbit | 1:1000 | Cell signaling | 9664S |
| Aβ | Rabbit | 1:500 | Cell signaling | #8243 |
| Aβ42 | Rabbit | 1:500 | Cell signaling | #12843 |
| p-tau (AT8) | Mouse | 1:500 | Invitrogen | MN1020 |
| total-tau | Mouse | 1:500 | Cell signaling | #4019 |
| CALNEXIN | Rabbit | 1:1000 | Cell signaling | #2679 |
| CHOP | Rabbit | 1:1000 | Cell signaling | #2895 |
| GSK3 αβ | Rabbit | 1:1000 | Cell signaling | #5676 |
| p-GSK3 αβ | Rabbit | 1:1000 | Cell signaling | #9331 |
| APP | Rabbit | 1:500 | Cell signaling | #2452 |
| PSEN1 | Rabbit | 1:1000 | Cell signaling | #5643 |
| BACE1 | Rabbit | 1:1000 | RnD systems | MAB391 |
| BACE1 | Mouse | 1:1000 | Cell signaling | #5606 |
| GAPDH | Rabbit | 1:1000 | Merck | MAB374 |
| β-actin | Mouse | 1:1000 | Cell signaling | 4967 |
