# Supplementary figures and images for: Zika virus infection accelerates Alzheimer’s disease phenotypes in brain organoids
Source: Cell Death Discov. 2022 Apr 2;8:153. doi: 10.1038/s41420-022-00958-x (PMC8976422; doi:10.1038/s41420-022-00958-x)

Original data: Western blot

Figure 2B

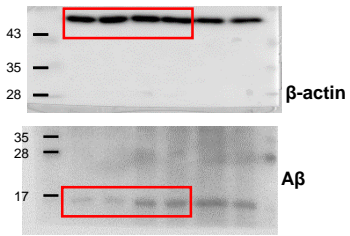

Figure 3C

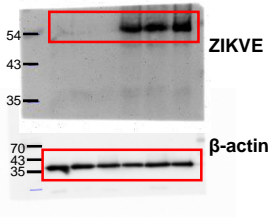

Figure 4E

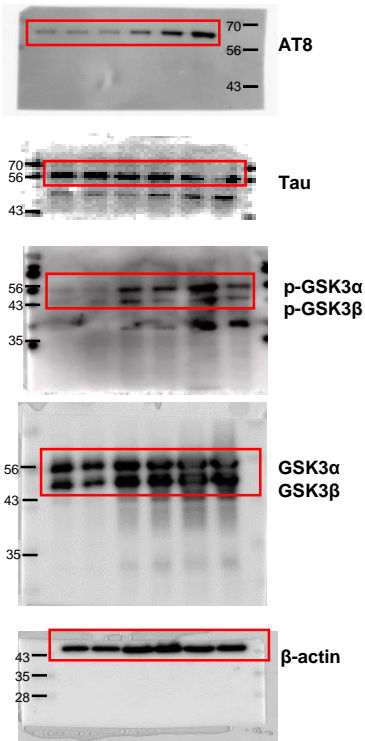

Figure 5A

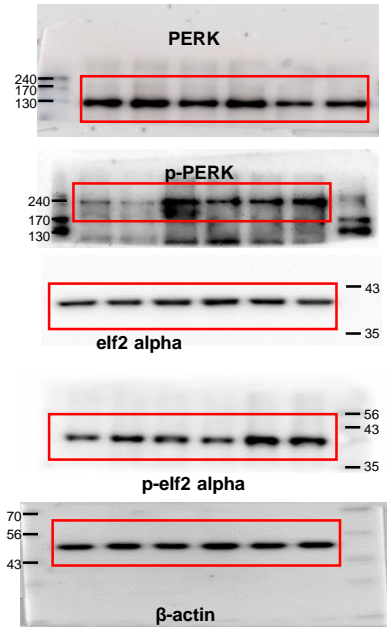

Figure 6A

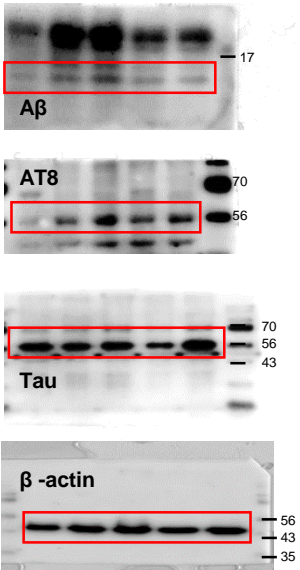

Figure 2F

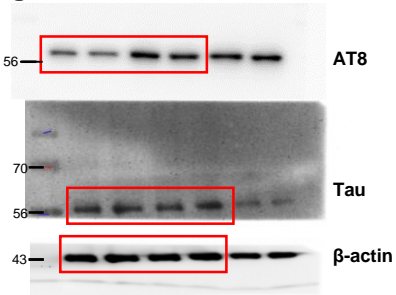

Figure 3I

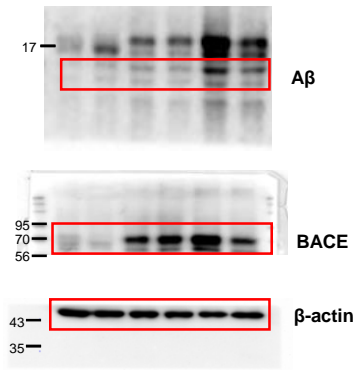

Figure 6I

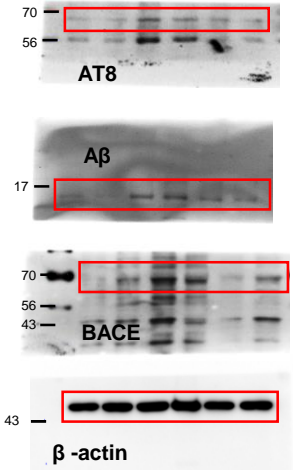

Supplement: Supplementary file 2 — Original Data File [file 41420_2022_958_MOESM2_ESM.pdf]
